# Embodied multimodal human-computer interaction for English language learning: integrating speech, gestures, emotion, and adaptive feedback

**DOI:** 10.3389/fnbot.2026.1920739

**Published:** 2026-10-14

**Authors:** Yao Tang

**Affiliations:** Jinshen College, Nanjing Audit University, Nanjing, China

**Keywords:** adaptive feedback generation, affective cognitive gating, cross modal transformer, embodied multimodal interaction, English language learning

## Abstract

Current English language learning systems are often limited by single-dimensional learning-state perception, insufficient modeling of affective-cognitive characteristics, and delayed or inflexible feedback regulation. To address these limitations, this study proposes an affective-cognitive gated cross-modal transformer (ACG-CMT) for embodied multimodal human-computer interaction in English language learning. The framework integrates learners’ speech, gestures, and affective responses as multimodal inputs. The speech encoder learns low-level acoustic-prosodic representations from Mel-spectral, energy, pitch/prosodic, and temporal patterns rather than receiving rubric-derived target scores; a spatiotemporal motion encoder captures gesture and posture dynamics; and an affective encoder models facial-action and vocal-affect cues. A cross-modal transformer captures temporal dependencies and contextual interactions among linguistic output, embodied behavior, and affective fluctuations. During fusion, an affective-cognitive gate adjusts modality-specific contributions using latent affective/cognitive context and pre-decision task context available at inference time, without access to ground-truth target labels. A multi-task decision module then supports learning-state recognition, affective-cognitive load estimation, and feedback-policy matching. ACG-CMT achieves an accuracy of 0.892, a macro-F1 of 0.884, an AUC of 0.941, and a difficult-state F1 of 0.877 for learning-state recognition; R^2^ = 0.836, RMSE = 0.084, and MAE = 0.068 for the operational affective-cognitive load reference index; and a feedback-policy matching rate of 0.861 with an average latency of 438 ms. These results indicate that the framework can integrate multimodal behavioral information for learner-state estimation and adaptive feedback, while the internal gating patterns are interpreted as model-level associations rather than causal psychological mechanisms.

## 1 Introduction

English language learning is undergoing a transition from traditional instructional paradigms centered on textbook-based teaching, classroom interaction, and outcome-oriented assessment toward intelligent human–computer interaction environments supported by speech recognition, computer vision, affective computing, and learning analytics technologies (An et al., 2025; Pratama et al., 2024; Shadiev and Yu, 2024). Recent developments in computer-assisted language learning (CALL) indicate that artificial intelligence-driven learning systems are increasingly capable of providing personalized assessment, adaptive learning resources, and interactive feedback. However, language learning remains a complex cognitive and behavioral process involving speech production, semantic processing, attention allocation, working memory regulation, and emotional control. Therefore, effective intelligent language learning systems should not only evaluate linguistic outcomes but also perceive learners’ dynamic behavioral states during interaction.

From the perspective of embodied cognition, language understanding and production are not exclusively dependent on abstract symbolic processing but emerge through continuous interactions among bodily experiences, sensory information, and environmental contexts (Garello et al., 2024; Lin and Yu, 2025; Macedonia, 2025). In second language learning scenarios, learners’ speech characteristics, body movements, facial expressions, and emotional responses provide important behavioral evidence regarding their learning engagement, cognitive difficulty, and interaction status (Hiver et al., 2024; Huang C. et al., 2024). Consequently, constructing an embodied multimodal human–computer interaction framework that integrates speech, gesture, emotion, and adaptive feedback has become an important direction for improving contextualized, interactive, and personalized language learning support.

Despite considerable progress, existing intelligent English learning systems still face several limitations in real-world learning environments. At the phonological level, pronunciation deviations, speech rate fluctuations, hesitation patterns, and prosodic variations are affected by factors such as native language transfer, task complexity, and psychological stress. Current computer-assisted pronunciation training systems mainly focus on pronunciation accuracy, phoneme alignment, and acoustic evaluation, but they often lack the capability to infer the underlying cognitive and affective states associated with language production (Bouchhioua, 2024; Guo, 2025; Issa and Hahn-Powell, 2025). As a result, speech-based evaluation alone may provide incomplete representations of learners’ actual learning conditions.

At the behavioral level, gestures, posture changes, and head movements represent important components of embodied communication, reflecting learners’ engagement, interaction intention, confidence, and comprehension difficulty. Recent studies on skeleton-based action recognition and spatiotemporal graph modeling have demonstrated the effectiveness of motion information for behavioral understanding (Gao et al., 2025; Li P. et al., 2025; Li Z. et al., 2025; Lovanshi and Tiwari, 2024). However, in educational applications, these bodily signals are often analyzed independently from linguistic performance, limiting the ability to explain how physical behaviors interact with language expression and learning processes.

At the affective level, emotional states such as anxiety, confusion, fatigue, and enjoyment significantly influence second language learning performance, attention allocation, and persistence during learning activities. Foreign language anxiety and emotional fluctuations have been shown to affect oral fluency and task performance, indicating the necessity of integrating affective information into intelligent learning systems (Bielak, 2025; Huang F. et al., 2024; Xin and Derakhshan, 2025; Zhao and Wang, 2025). Although affective computing approaches based on facial expression analysis, speech emotion recognition, and multimodal emotional cues have improved emotion perception capabilities, many existing approaches remain focused on emotion classification rather than establishing a stable relationship between affective states, cognitive load, language performance, and adaptive instructional decisions (Haq et al., 2024; Kopalidis et al., 2024; Zhang and Tan, 2024).

Furthermore, adaptive feedback remains a critical challenge in intelligent language learning systems. Conventional feedback mechanisms are frequently based on predefined templates or isolated error detection results, making it difficult to dynamically adjust feedback type, timing, and intensity according to learners’ immediate performance and psychological state. Previous research has demonstrated that automated corrective feedback and regulation-oriented feedback can influence learning achievement, motivation, and cognitive load; however, effective feedback requires accurate perception of learners’ current interaction states (Feng and Yang, 2025; Liu C. C. et al., 2025; Liu Y. et al., 2025; Yildiz Durak, 2024). Therefore, a more comprehensive framework is needed to connect multimodal perception with adaptive feedback generation.

To address these limitations, existing research has explored several technological directions, including automatic speech recognition, behavioral analysis, affective computing, and multimodal deep learning. Speech-based intelligent correction models have achieved considerable progress in grammar correction, pronunciation assessment, and language error detection, but they mainly rely on linguistic signals and lack joint modeling of embodied behavior and emotional states (Chen and Xiao, 2024; Raju et al., 2024; Xiao and Yin, 2024). Meanwhile, gesture recognition approaches based on pose estimation and spatiotemporal modeling provide valuable information about learner behavior but generally remain separated from linguistic and affective processes (Gao et al., 2025; Li P. et al., 2025; Li Z. et al., 2025).

Multimodal deep learning methods attempt to integrate speech, visual, and textual information into unified representations. Transformer-based architectures have recently demonstrated strong capabilities in cross-modal interaction modeling; however, existing approaches often rely on early fusion, late fusion, or relatively static attention mechanisms, which may not sufficiently address temporal misalignment among modalities, dynamic changes in modal importance, and affective-cognitive modulation during learning interactions (Faria et al., 2025; Khan et al., 2025; Tian et al., 2024). Recent cross-modal Transformer studies combining facial expression, EEG, and other physiological signals have further demonstrated the potential of Transformer-based architectures for complex multimodal representation learning (Feng et al., 2025; Li P. et al., 2025; Li Z. et al., 2025). Nevertheless, these approaches primarily focus on affective state recognition and multimodal representation enhancement, while the integration of learner state perception, cognitive load estimation, and adaptive feedback generation remains insufficiently explored.

Therefore, current intelligent English learning systems still face three major challenges: (1) incomplete perception of learners’ multimodal interaction states; (2) insufficient integration of affective-cognitive information into representation learning; and (3) limited capability to transform perceived learner states into adaptive instructional feedback. Addressing these challenges requires a framework that can dynamically integrate speech, gesture, emotional responses, and contextual interaction information while maintaining interpretability and adaptability.

To address incomplete multimodal state recognition, insufficient affective-cognitive characterization, and lagging feedback regulation in English learning systems, this paper proposes the Affective-Cognitive Gated Cross-modal Transformer (ACG-CMT). Three dedicated encoding branches generate acoustic-prosodic, bodily-engagement, and affective representations from synchronized multimodal inputs. A temporal-alignment mechanism unifies sequence scales, and a cross-modal Transformer establishes bidirectional associations among speech, motion, and affective fluctuations. The affective-cognitive gating unit then reallocates modality weights from latent representations available during inference rather than from ground-truth target labels. A multi-task head maps the fused representation to learning-state recognition, affective-cognitive load estimation, and feedback-policy matching. Relative to conventional multimodal Transformers, the contribution of ACG-CMT is therefore framed as state-dependent fusion and joint task optimization. The resulting gating weights provide model-level indicators of changing modality reliance across learning states; they are not presented as direct causal explanations of human cognitive mechanisms.

## 2 Embodied interaction model

### 2.1 System overall framework design

An embodied multimodal human-computer interaction system for English language learning takes learners’ synchronized speech signals, gesture/pose signals, and affective responses as its basic inputs. The framework is organized around multimodal perception, cross-modal state representation, affective-cognitive gating fusion, and adaptive feedback generation. Importantly, the predictor set is separated from the variables used to construct supervision labels. The speech branch therefore receives raw or low-level acoustic-prosodic representations (e.g., Mel-spectral, energy, pitch/prosodic, and temporal patterns) rather than rubric-derived pronunciation-accuracy scores; the gesture branch receives pose/keypoint dynamics; and the affective branch receives facial-action and vocal-affect features. Variables used directly to construct a target label are not concatenated back into the predictor vector for that target. ACG-CMT establishes dynamic cross-modal connections within a unified time window and adjusts modality contributions through the gating mechanism. The overall structure is shown in Figure 1. Figure 1 is a schematic illustration of the implemented model configuration.

**Figure 1:**
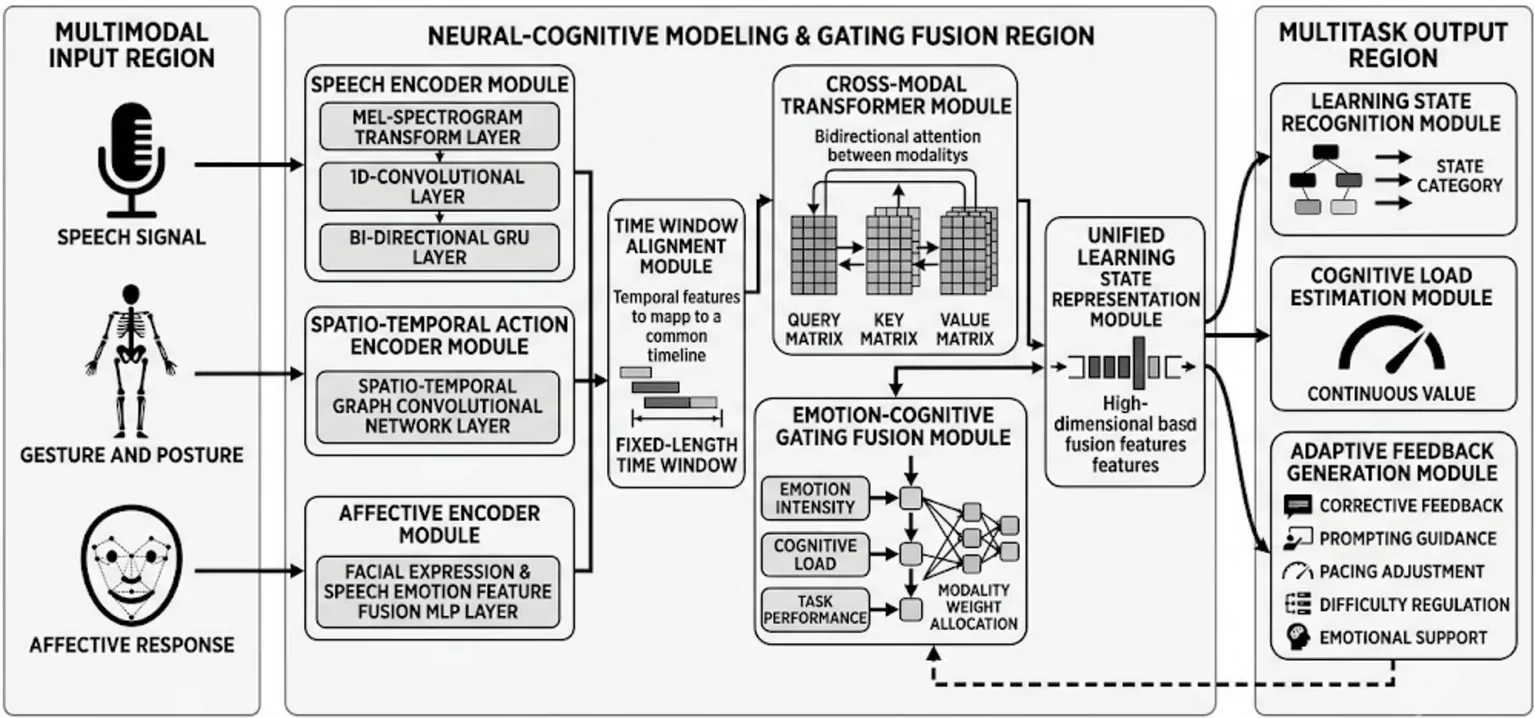
An embodied multimodal interaction framework for English language learning, developed by ACG-CMT.

In the system input layer, the learner’s multimodal behavior is defined as a synchronous sequence of speech, gestures, and emotional responses, as shown in Equation 1:

$$X=\{X^{s},X^{g},X^{a}\}$$ (1)

In Equation 1, X represents the learner’s overall multimodal input; Xs denotes the speech sequence containing acoustic information such as spectral/prosodic dynamics, speech timing, and pauses; Xg denotes the gesture/posture sequence containing keypoint and motion trajectories; and Xa denotes the affective sequence containing facial-action/expression dynamics and vocal-affect features. Xa does not contain human-assigned emotional-state labels, and none of the three input streams contains the supervision targets for learning state, the operational load reference index, or feedback policy. The three modalities correspond to the same learning-behavior segment on the temporal axis, providing synchronized inputs for subsequent cross-modal modeling.

During model execution, ACG-CMT feeds the three predictor streams into independent encoders to obtain speech, gesture, and affective representations, after which a cross-modal Transformer models their temporal dependencies. The fused representations enter the affective-cognitive gate, whose weights are conditioned on latent affective/cognitive context and pre-decision task context derived from information available at inference time. The supervision targets for learning state, the operational load reference index, and feedback policy are not supplied to the gate. The resulting representation supports learning-state recognition, affective-cognitive load estimation, and feedback-policy matching. This design is motivated by embodied-cognition perspectives in that it jointly models linguistic output, bodily engagement, and affective behavior; however, the architecture should not be interpreted as a direct mechanistic implementation or validation of embodied-cognition theory.

### 2.2 Embodied feature encoding of voice, gestures, and emotions

Speech, gestures, and emotional responses correspond to language output, bodily engagement, and affective regulation during English learning. To ensure consistent representation scales across the three modalities, the original multimodal streams are transformed into hidden feature sequences with uniform temporal length and dimension. The speech branch is driven by acoustic-prosodic information extracted from the waveform/Mel representation, including spectral dynamics, energy, pitch/prosodic rhythm, and temporal patterns; rubric-derived pronunciation scores and other variables used directly in target construction are not supplied as model inputs. The gesture branch extracts keypoint trajectories, gesture amplitude, posture stability, and movement continuity. The affective branch integrates facial action/expression dynamics and vocal-affect cues. This separation is used to reduce the risk that a target can be reconstructed trivially from variables used to define that same target.

The speech feature encoding adopts a combination of Mel spectrum transform, one-dimensional convolution and bidirectional gated recurrent units to map continuous speech signals into speech hidden layer representations, as shown in Equation 2:

$$H^{s}=f_{s}(X^{s};\theta_{s})={\text{BiGRU}}_{s}({\mathrm{CNN}}_{s}(\mathrm{Mel}(X^{s})))$$ (2)

In Equation 2, H^s^ represents the hidden layer representation of the speech modality; X^s^ represents the input speech sequence; f_s_(·) represents the speech coding function; θ_s_ represents the trainable parameters of the speech encoder; Mel(·) represents the Mel spectral transform; CNN_s_(·) represents a one-dimensional convolutional layer; and BiGRU_s_(·) represents a bidirectional gated recurrent unit. This coding process is used to extract temporal features from articulation, prosody, and pause structures.

Gesture pose feature encoding uses a spatiotemporal graph convolutional network to model the sequence of human key points. The key points are used as graph nodes, and the connection relationships of body joints and the motion relationships in adjacent time segments are used as graph edges to represent the learner’s movement changes and body participation status in the process of English expression, as shown in Equation 3:

$$\begin{array}{c} H^{g}=f_{g}(X^{g};\theta_{g})=\mathrm{ST}-{\mathrm{GCN}}_{g}(\text{Pose}(X^{g})) \end{array}$$ (3)

In Equation 3, H^g^ represents the hidden layer representation of the gesture pose modality; X^g^ represents the video pose input sequence; f_g_(·) represents the gesture pose encoding function; θ_g_ represents the trainable parameters of the gesture pose encoder; Pose(·) represents the human keypoint extraction process; and ST-GCN_g_(·) represents the spatiotemporal graph convolutional network. This encoding process is used to extract gesture amplitude, pose changes, and interaction participation features.

Emotional feature encoding maps facial expression features and speech emotion features to a unified emotional space. Facial expression features reflect the intensity of expression and emotional tendency, while speech emotion features reflect the emotional information in changes in tone, energy, and rhythm. After feature connection and nonlinear mapping, the two types of emotional cues form an emotional latent layer representation, as shown in Equation 4:

$$\begin{array}{c} H^{a}=f_{a}(X^{a};\theta_{a})={\mathrm{MLP}}_{a}([F^{\text{face}};F^{\text{voice}}]) \end{array}$$ (4)

In Equation 4, H^a^ represents the hidden layer representation of the emotional modality; X^a^ represents the emotional modality input; f_a_(·) represents the emotional encoding function; θ_a_ represents the trainable parameters of the emotional encoder; F^face^ represents facial expression features; F^voice^ represents voice emotion features; [;] represents the feature connection operation; and MLPa(·) represents the multilayer perceptron mapping layer. This encoding process is used to form the learner’s emotional state and emotional intensity representation.

To visually represent the differences in representation among the three modalities of speech, gesture/pose, and emotion after feature encoding, a two-dimensional dimensionality reduction visualization was performed on the speech encoding features, gesture/pose encoding features, and emotion encoding features. In Figure 2, each point represents a window-level learning sample, and different colors represent different learning state categories. This visualization result is used to observe the representational ability of the three types of encoders to the differences in learning states and to provide intermediate feature basis for subsequent cross-modal temporal correlation modeling, as shown in Figure 2. The distributions in Figure 2 are real experimental outputs obtained by applying t-distributed stochastic neighbor embedding to the encoder features of all test set samples.

**Figure 2:**
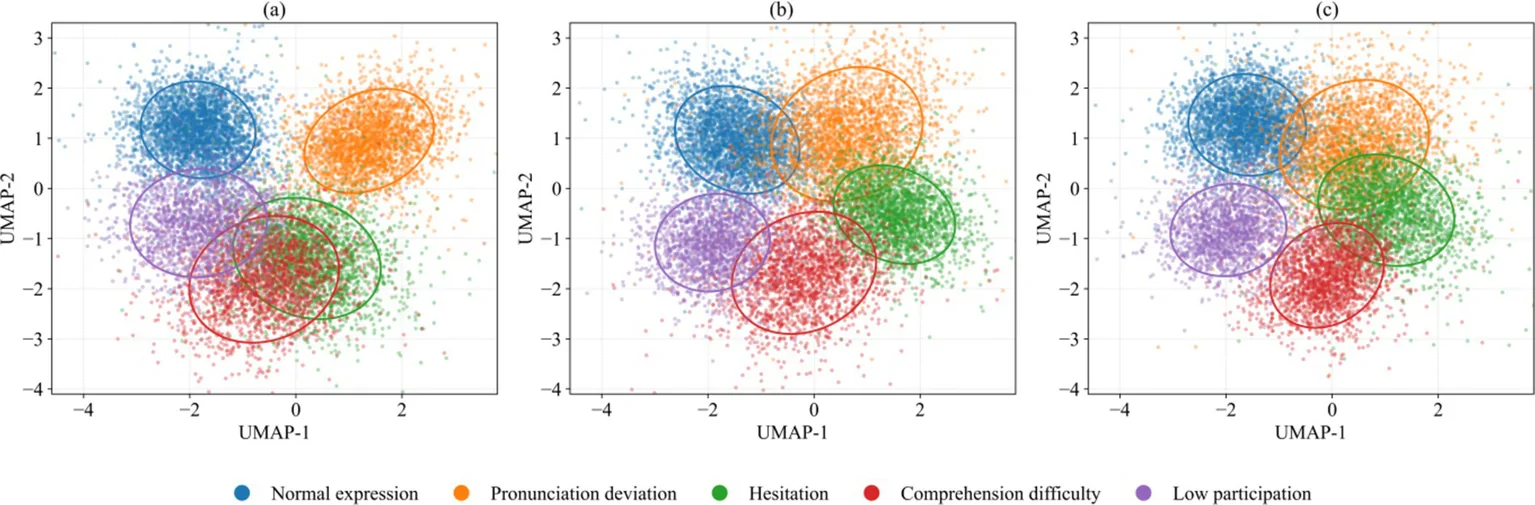
Distribution of speech, gesture, and emotion feature encoding results. **(a)** Speech feature distribution. **(b)** Gesture-posture feature distribution. **(c)** Affective feature distribution.

As shown in Figure 2, the three modality encoders exhibit category clustering and partial boundary separation in the two-dimensional visualization, indicating that speech, gesture, and affective streams contain discriminative information about learning states. The speech representation shows stronger separation for pronunciation-related states, the gesture representation reflects hesitation and participation changes, and the affective representation differentiates comprehension difficulty, low participation, and affective fluctuation. Because these patterns are based on a dimensionality-reduction visualization, they are interpreted descriptively rather than as a formal test of statistical significance. The non-identical distributions across modalities also illustrate their complementary information content and motivate subsequent cross-modal modeling.

### 2.3 Cross-modal temporal alignment and correlation modeling

Speech, gestures, and emotional responses naturally differ in sampling frequency, temporal duration, and dynamic variation patterns. Consequently, directly feeding heterogeneous multimodal sequences into a cross-modal Transformer may result in temporal misalignment, where corresponding learning behaviors are represented at inconsistent positions across modalities. Such misalignment can weaken cross-modal dependency learning and reduce the model’s ability to capture the temporal relationships among learners’ verbal expression, embodied behaviors, and affective changes. To address this issue, this study adopts a fixed-length temporal window as an alignment reference, mapping speech, gesture, and affective representations onto a unified temporal axis while preserving the intrinsic sequential characteristics of each modality (Jin et al., 2024; C. C. Liu et al., 2025; Liu Y. et al., 2025).

The modal temporal alignment process takes the hidden layer representation obtained in Section 2.2 as input and generates modal sequences of consistent length through unified resampling and positional encoding, as shown in Equation 5:

$$\begin{array}{c} {\tilde{H}}^{m}=\text{Align}(H^{m},T)=\mathrm{PE}(\text{Interp}(H^{m},\tau^{m}\to T)),m\in \{s,g,a\} \end{array}$$ (5)

In Equation 5, ${\tilde{H}}^{m}$ represents the hidden layer representation after modality m alignment; H^m^ represents the original encoded representation of modality m; T represents the uniform time window length; τ^m^ represents the original time index of modality m; Interp(·) represents the time interpolation or resampling operation; PE(·) represents the positional encoding operation; m∈{s,g,a} corresponds to speech, gesture, and emotion modalities, respectively. This operation only adjusts the sequence length and temporal position of different modalities without changing the semantic dimension of the original features, so that subsequent attention calculations can simultaneously preserve cross-modal synchronization relationships and intramodal order information.

After completing temporal alignment, this paper employs a cross-modal attention mechanism to establish dynamic dependencies between different modalities. For the target modality m, the model generates a query matrix based on this modality and a key matrix and a value matrix based on the associated modality n. The attention intensity of the target modality to the associated modality is calculated by the similarity between the query and the key. The cross-modal attention calculation is shown in Equation 6:

$$\begin{array}{c} \text{Attn}(Q_{m},K_{n},V_{n})=\text{Softmax}(\frac{Q_{m}K_{n}^{T}}{\sqrt{d_{k}}})V_{n},\;m\neq n \end{array}$$ (6)

In Equation 6, $\text{Attn}(Q_{m},K_{n},V_{n})$ represents the cross-modal attention output of modality m to modality n; Q_m_ represents the query matrix of the target modality m; K_n_ and V_n_ represent the key matrix and value matrix of the associated modality n, respectively; d_k_ represents the dimension of the key vector; Softmax(·) is used to normalize the attention weights between different time segments; m ≠ n indicates that the attention calculation occurs between different modalities. This calculation enables speech pauses, gesture changes, and emotional fluctuations to form associations within the same learning segment, avoiding the weakening of modal relationships caused by relying solely on static feature splicing.

In the multimodal interaction modeling stage, ACG-CMT aggregates the multi-directional attention results between different modalities to form a contextual representation of each modality after absorbing external information, as shown in Equation 7:

$$\begin{array}{c} C^{m}=\text{LayerNorm}({\tilde{H}}^{m}+\sum_{n\neq m}\text{Attn}(Q_{m},K_{n},V_{n})) \end{array}$$ (7)

In Equation 7, C^m^ represents the cross-modal contextual representation of modality m; the aligned representation of modality m is used as the residual term; the attention outputs from modalities other than the target modality are aggregated to provide cross-modal contextual information; and LayerNorm(·) denotes the layer normalization operation. The equation performs residual fusion between the target modality representation and the contextual information provided by other modalities, enabling the model to absorb cross-modal information while retaining the original modal features.

The attention response output of the cross-modal Transformer layer is visualized and analyzed. In the figure, the three modalities of speech, gesture, and emotion are organized into cross-modal block attention matrices according to time segments to present the response intensity and local attention regions between different modal segments. At the same time, the time delay response curves of the three cross-modal relationships of speech-gesture, speech-emotion, and gesture-emotion are extracted to observe the synchronization relationship and delay characteristics between modalities. The results are shown in Figure 3. The attention matrices and the lag response curves in Figure 3 are computed from the trained ACG-CMT on the test set rather than drawn as conceptual illustrations.

**Figure 3:**
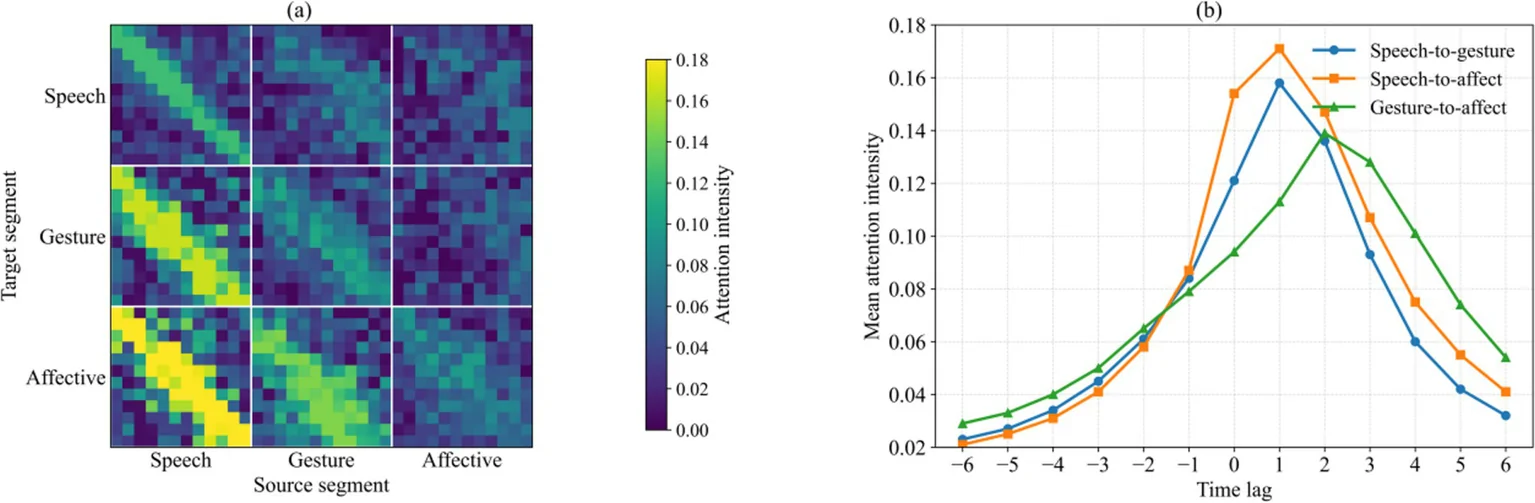
Cross-modal attention association and temporal lag response features. **(a)** Cross-modal block attention matrix. **(b)** Temporal lag response of cross-modal attention.

As shown in Figure 3, ACG-CMT exhibits non-uniform cross-modal attention allocation rather than simple average fusion. Speech-driven gesture and affective response regions show localized concentration, whereas gesture-to-affect attention is distributed across a wider temporal range; the reverse blocks are generally weaker and more dispersed. Figure 3b further shows different lag-response profiles for speech-gesture, speech-affect, and gesture-affect relationships. These attention patterns are descriptive properties of the trained model and indicate that the model captures temporally structured cross-modal associations. They should not be interpreted as formal statistical significance or as evidence of causal directional influence among the human behavioral modalities.

### 2.4 Affective-cognitive gating fusion mechanism

After temporal alignment and cross-modal correlation modeling, speech, gesture, and affective modalities obtain contextual representations enriched by information from other modalities. However, the contribution of each modality may vary significantly under different learning conditions. For example, pronunciation-related difficulties may be more strongly reflected through speech features, whereas hesitation, reduced engagement, or emotional fluctuations may require greater reliance on gesture and affective information. Therefore, fixed-weight fusion strategies are insufficient for representing state-dependent changes in modality importance during interactive learning scenarios. Recent studies on multimodal intelligent educational systems and adaptive interaction have emphasized the importance of dynamically integrating emotional and cognitive-related behavioral signals to improve personalized feedback generation (Gong, 2026; Kong et al., 2025).

Based on this observation, this study introduces an affective-cognitive gating fusion mechanism within ACG-CMT. Unlike conventional fusion approaches that maintain relatively stable modality contributions after training, the proposed gating mechanism incorporates observable affective intensity, cognitive load estimation, and task performance indicators into the modality weighting process. This design enables the model to dynamically adjust the contributions of speech, gesture, and affective information according to learners’ current interaction states, thereby improving the adaptability and interpretability of multimodal learning state representation.

The affective-cognitive gating unit takes the cross-modal contextual representations Cs, Cg, and Ca as its main inputs and combines them with an affective context representation E, a latent cognitive-context representation L, and an online task-context representation P to generate normalized gating weights for the three modalities, as shown in Equation 8. Here, L is an internal latent representation computed from hidden features during the forward pass; it is not the cognitive-load reference label yload and does not contain NASA-TLX, reaction time, pause ratio, task difficulty, or any other component used directly to construct yload. Likewise, P contains only information available to the system before the feedback decision and excludes teacher feedback labels and post-feedback outcomes.

$$\begin{array}{c} Z=\text{Softmax}(W_{z}[C^{s};C^{g};C^{a};E;L;P]+b_{z}) \end{array}$$ (8)

In Equation 8, Zs, Zg, and Za denote the normalized weights of the speech, gesture, and affective modalities; Cs, Cg, and Ca denote their cross-modal contextual representations; E denotes the affective context embedding; L denotes the internally estimated latent cognitive-context embedding; and P denotes pre-decision task context. Wz and bz are the trainable parameters of the gating layer and Softmax(·) normalizes the three modality weights. The distinction between L and the supervised load target yload is critical: no supervised operational load reference-index value or component used to construct that index is fed into the gating unit. Thus, the gate is conditioned on representations available at inference time rather than on target information.

After obtaining the gating weights, the model performs weighted fusion of the three types of cross-modal context representations to form a unified learning state representation, as shown in Equation 9:

$$\begin{array}{c} H=\text{LayerNorm}(Z_{s}⊙C^{s}+Z_{g}⊙C^{g}+Z_{a}⊙C^{a}) \end{array}$$ (9)

In Equation 9, H represents the fused learning state representation; Z_s_ ⊙ C^s^, Z_g_ ⊙ C ^g^, and Z_a_ ⊙ C^a^ represent the weighted representations of speech, gestures, and emotional modalities, respectively; ⊙ represents element-wise multiplication; and LayerNorm(·) represents layer normalization. This fusion process integrates language output, bodily engagement, and emotional cognitive states into the same representation space, providing input for learning state recognition, emotional-cognitive load estimation, and feedback strategy matching.

This gating fusion mechanism transforms multimodal fusion from static feature integration to state-driven dynamic representation updates, enabling the model to form differentiated modal dependencies under different learning states. To demonstrate the moderating effect of the affective-cognitive gating unit on the contributions of the three modalities, this paper extracts the gating weights under different learning states and compares the response changes of speech, gesture, and emotion modalities. The gating weight responses are shown in Figure 4.

**Figure 4:**
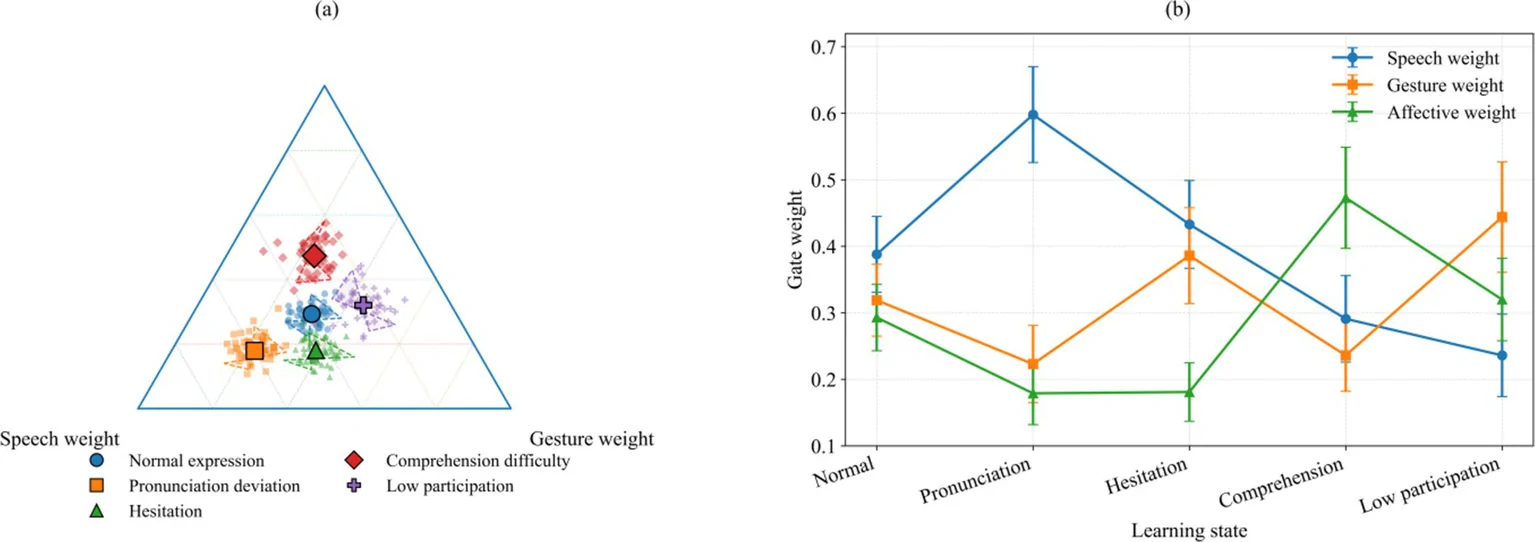
Affective-cognitive gating weighted response characteristics. **(a)** Gated modality weight simplex with state-level distributions. **(b)** Gated modality response profile.

As shown in Figure 4, gating-weight distributions vary across English learning states. Normal-expression samples lie closer to the trimodal balance region; pronunciation-deviation samples shift toward the speech-weight direction; hesitation samples occupy an intermediate speech-gesture region; comprehension-difficulty samples shift toward the affective direction; and low-engagement samples rely relatively more on gesture/affective information. Figure 4b shows the same state-dependent trend in the modality response profiles. These differences are presented as descriptive model-internal patterns rather than inferentially significant effects. They provide a *post hoc* indication of how the trained gate reallocates modality reliance across observed states, not a causal explanation of the learner’s underlying cognitive process.

### 2.5 Adaptive feedback generation and joint optimization

After affective-cognitive gating fusion, the model obtains a unified representation H containing learned information about language output, bodily engagement, affective context, latent cognitive context, and pre-decision task context. These are model representations inferred from the input streams; H does not contain the supervised operational load reference-index target or the teacher feedback label. The adaptive feedback module takes H as input and produces the learning-state category, affective-cognitive load estimate, and feedback-policy distribution through the multi-task decision head. The three tasks share H, enabling feedback generation to be driven by information available from the learner’s current multimodal interaction rather than by target labels.

To achieve synergistic optimization of learning state recognition, affective-cognitive load estimation, and feedback strategy matching, this paper incorporates the three types of task losses and model regularization terms into the training objective, as shown in Equation 10:

$$\begin{array}{c} L=\lambda_{1}L_{\text{state}}+\lambda_{2}L_{\text{load}}+\lambda_{3}L_{\text{feedback}}+\lambda_{4}{\left\|\Theta \right\|}_{2}^{2} \end{array}$$ (10)

In Equation 10, L represents the joint optimization objective of the model; L _state_ represents the cross-entropy loss for learning state recognition, used to constrain ${\hat{y}}_{\text{state}}$ the difference between the predicted state category and the true state label y_state;_ L_load_ represents the mean squared error loss ${\hat{y}}_{\text{load}}$ for affective-cognitive load estimation, used to constrain the error between the predicted load value and the operational load reference-index target y_load;_ L _feedback_ represents the cross-entropy loss for feedback policy matching, used to constrain the consistency between the predicted feedback policy ${\hat{y}}_{\text{feedback}}$ and the teacher or expert labeled policy y _feedback_; λ1, λ2, λ3, and λ4 are loss weight coefficients; Θ represents all trainable parameters of ACG-CMT; ${\left\|\Theta \right\|}_{2}^{2}$ represents the L_2_ regularization term. This joint objective coordinates different learning tasks in a weighted manner, ensuring consistency between state recognition, affective-cognitive load estimation, and feedback policy generation.

After training, the system generates a fused representation H based on real-time input of speech, gestures, and emotional responses. The feedback decision module then outputs feedback results that match the current learning state. This process connects multimodal perception, affective-cognitive gating, and adaptive feedback decision-making into a unified model, providing a methodological foundation for subsequent learning state recognition, affective-cognitive load estimation, feedback policy matching, and interaction efficiency evaluation.

## 3 Experimental design and data configuration

### 3.1 Experimental subjects and multimodal data acquisition

This human-participant study collected data from 96 non-English-major undergraduate students aged 18–23 years, all of whom had at least six consecutive years of English learning experience and normal visual and auditory functions. The study was conducted in accordance with the institutional requirements for educational research at Nanjing Audit University Jinshen College, Nanjing, China. The study protocol and multimodal data-collection procedures were reviewed and approved by the relevant institutional authority before participant recruitment. No separate ethics approval number was assigned under the institutional procedures applicable to this study. Before participation, all learners were informed of the research purpose, experimental procedures, categories of data to be collected, and intended use of the research data, and written informed consent was obtained from every participant. Participation was voluntary. The collected audio, video, facial-expression, posture and gesture sequences, and interaction logs were used solely for the purposes of this study. All samples were anonymized before model training and analysis to protect participants’ identities and behavioral privacy.

The experimental data acquisition process was conducted in a quiet indoor environment. Speech signals were acquired using a headset microphone at a sampling rate of 16 kHz to record pronunciation, speech rate, pauses, and prosodic variations. Video signals were acquired using a front-facing high-definition camera at a frame rate of 30 fps to record facial expressions, head posture, upper limb movements, and gesture trajectories. Interaction logs were automatically recorded by the learning system, saving task type, response time, system feedback, teacher annotations, and task completion status. Equipment calibration was performed before acquisition, and during acquisition, camera angle, microphone distance, task presentation method, and ambient lighting were kept relatively consistent to reduce the impact of environmental noise and equipment differences on multimodal feature extraction.

The supervision set consists of learning-state labels, affective-state information, an affective-cognitive load reference index, and feedback-strategy labels. Learning-state labels were assigned by human annotators using a predefined rubric covering pronunciation quality, fluency, semantic completeness, hesitation/comprehension difficulty, and participation; the rubric criteria were used for annotation and were not copied into the model as target scores. The affective-cognitive load variable was constructed by combining NASA-TLX, reaction time, pause ratio, and task difficulty after normalizing each component to the 0–1 range. NASA-TLX serves as the subjective workload anchor, reaction time and pause ratio provide complementary behavioral manifestations of processing difficulty, and task difficulty provides task-context calibration. Consistent with the original annotation procedure, the relative weighting was jointly determined by two English-teaching researchers and one learning-analytics researcher based on the intended meaning of the indicators. Because the study did not include an independent physiological criterion, this quantity is referred to throughout the revision as an operational composite reference index rather than a physiological or mechanistic ‘ground truth’. Feedback-strategy labels were independently assigned by English teachers from five categories: error correction, prompting, pacing adjustment, difficulty adjustment, and emotional support. Each categorical sample was independently reviewed by two annotators, with low-agreement cases adjudicated by a third teacher; Cohen’s kappa was used to summarize categorical agreement. This design provides content-based and expert-review support for the labels, while the absence of an independent physiological criterion for the composite load index is explicitly acknowledged as a limitation. The experimental data sources and variable configurations are shown in Table 1. Crucially, this composite has not been independently established for criterion, convergent, or physiological validity, and it is not treated as a validated ground-truth measure of latent cognitive load. All load-estimation metrics in this study therefore quantify prediction of, and agreement with, this operational reference index only.

**Table 1:** Experimental data sources and variable configuration table.

Data type	Source	Data scale	Variables	Values or dimensions
Speech	Head-mounted microphone, 16 kHz	96 learners; 3,840 segments; 11.6 h	Mel spectrogram; speech rate; pause ratio; energy; prosody	80 × 500; 2.84 words/s; 0.216; 0.637
Gesture posture	Frontal video, 30 fps; MediaPipe Pose	3,840 segments; 1,252,800 frames	Keypoints; gesture amplitude; motion continuity; posture stability	33 × 3 × 150; 0.418; 0.693; 0.741
Affective features	OpenFace; acoustic emotion features	1,252,800 frames; 12,864 windows	Facial AUs; expression intensity; vocal emotion; emotional stability	35 dims; 26 dims; 0.462; 0.684
Interaction logs	Learning system records	3,840 logs; 12,864 windows	Task type; response duration; feedback time; completion status	4 task types; 10.9 s; 438 ms
Learning state labels	Annotated by two raters	12,864 labels	Normal; pronunciation deviation; hesitation; comprehension difficulty; low participation	3,426; 2,864; 2,547; 2,191; 1836; κ = 0.824
Affective-cognitive load reference index	NASA-TLX and behavioral indicators	12,864 labels	Load score; load level; response duration; pause ratio	0–1; mean = 0.536; low = 3,868; medium = 5,703; high = 3,293
Feedback labels	Annotated by English teachers	12,864 labels	Correction; prompt; rhythm adjustment; difficulty regulation; emotional support	3,128; 2,764; 2,419; 2,217; 2,336; κ = 0.812

Clarification on predictor-label separation: Table 1 inventories variables recorded or derived during the experiment and therefore includes some variables used only for label construction or descriptive analysis. It should not be read as the final predictor list for every task. For the corresponding prediction head, variables that directly enter target construction (including rubric-derived pronunciation scores; NASA-TLX; reaction time; pause ratio; task-difficulty terms used in the load composite; and teacher-assigned feedback labels) are excluded from the model input. The neural encoders operate on synchronized acoustic, pose, facial-action, and vocal-affect streams, and all preprocessing parameters are estimated without using test-set labels.

### 3.2 Data preprocessing and sample construction

To ensure that speech, gestures, facial expressions, and interaction logs participate in model training on the same timescale, this paper uses learning task segments as the basic unit for data preprocessing. Speech data undergoes denoising, silence detection, volume normalization, and segmentation to retain effective speech intervals relevant to English expressions. Video data undergoes frame rate unification, image cropping, and key region localization to extract human posture, facial expressions, and head posture features. Interaction logs are calibrated based on task start time, response end time, and feedback trigger time to determine the task type, response duration, and feedback label for each sample.

In the speech preprocessing stage, the raw audio is uniformly converted into a 16 kHz mono signal, and spectral subtraction and short-time energy constraints are used to reduce the impact of environmental noise. Speech activity detection is used to divide the effective vocal intervals, and the segmented speech segments are used to extract Mel spectrum, pronunciation duration, pause ratio, speech rate, energy changes, and prosodic features. Each speech segment is truncated or padded according to a fixed time window to meet the input length requirements of the ACG-CMT speech encoder, while reducing the interference of invalid silences on the recognition of the learning state. The Mel spectrum is computed with 80 filter banks, a frame length of 25 ms, and a frame shift of 10 ms, and each speech segment is fixed to 5 s with zero-padding applied to shorter segments.

In the video preprocessing stage, this paper uses MediaPipe Pose to extract key points for the head, shoulders, elbows, wrists, and hands, forming a continuous pose sequence. OpenFace is used to extract facial motion units, eye opening and closing, mouth movements, and head orientation features. After outlier removal, coordinate normalization, and smoothing filtering, the key point sequence is used to characterize gesture amplitude, motion continuity, pose stability, and body involvement. Both pose coordinates and facial features are normalized using relative positions to reduce the impact of individual body size, camera distance, and facial scale differences on model training. MediaPipe Pose version 0.10.14 is used to extract 33 body keypoints per frame, and OpenFace version 2.2.0 is used to extract facial action units, eye and mouth states, and head orientation, yielding 35 facial feature dimensions per frame.

In the label preprocessing stage, learning-state labels, affective information, the affective-cognitive load reference index, and feedback-strategy labels are mapped to the same task segment/window identifiers. Emotional labels are temporally smoothed to reduce single-frame fluctuations. The load reference index combines normalized NASA-TLX, reaction time, pause ratio, and task difficulty using the expert-weighting procedure described above. This composite is used only as supervision for the load-estimation task; its constituent variables are not directly supplied to the load-estimation head or the gating unit. The weighting reflects a content-validity rationale—subjective workload anchored by NASA-TLX, complemented by behavioral delay/hesitation and task context—rather than a claim of criterion validity against physiological cognitive load. Feedback labels are aligned with the system trigger time. Samples are removed when missing speech exceeds 30%, valid pose frames are below 70%, or annotation conflicts remain unresolved after adjudication. The present study does not claim that the expert-weighted combination is uniquely optimal; alternative weighting schemes were not independently validated in this dataset. Accordingly, the composite is retained as an operational supervision target, and the sensitivity of conclusions to alternative weighting schemes remains an explicit limitation for future validation.

Sample construction used a 5-s window with a 2.5-s step (50% overlap). The 3,840 observations reported at the segment level are task-level multimodal interaction segments; they are not identical to the final model instances. For a segment of duration di > 5 s, successive 5-s windows were generated every 2.5 s; segments shorter than 5 s produced one padded and masked window. Applying this procedure to the 3,840 task segments yielded 12,864 synchronized model windows, corresponding to an average of 3.35 windows per task segment (12,864/3,840). Each model window contains synchronized speech, gesture/pose, and affective sequences together with the applicable supervision labels. Adjacent windows from the same segment share up to 2.5 s of content and are therefore correlated. Consequently, 12,864 is the number of computational windows, not the number of statistically independent observations; the principal independent clustering unit for generalization is the learner, with task segment as an intermediate clustering level.

The overlap-induced correlation has two implications for statistical interpretation. First, the effective sample size is smaller than the raw window count and depends on the within-segment/within-learner correlation, so a single iid-based effective-N value is not asserted. Second, confidence intervals or hypothesis tests that resample individual windows would be anti-conservative because they would treat correlated windows as independent. We therefore use learner-level partitioning to prevent train-test contamination and interpret the reported window metrics as predictive performance estimates, while avoiding inferential claims based on 12,864 independent observations.

### 3.3 Model training, comparative experiments, and evaluation metrics

The data are divided into training, validation, and test subsets in a 7:1:2 ratio using learner-level grouping: every window generated from a given learner is assigned to only one subset. This grouping is performed before model fitting, hyperparameter selection, and test evaluation, and prevents adjacent/overlapping windows from the same learner from appearing across training and test sets. Preprocessing statistics used for model input normalization are estimated from the training subset and then applied to validation/test data. The subsets are balanced as far as possible with respect to English proficiency, task type, and learning-state distribution. ACG-CMT is implemented in PyTorch 2.1.0 with CUDA 12.1 under Python 3.10 and trained on a single NVIDIA RTX 4090 GPU with 24 GB of memory; one complete run takes approximately 3.5 h. AdamW uses beta coefficients of 0.9 and 0.98, cosine learning-rate decay, and a five-epoch warm-up. Batch size is 32, the maximum number of epochs is 100, the initial learning rate is 0.0001, and weight decay is 0.0005. The cross-modal Transformer contains 4 layers, 8 attention heads, a 256-dimensional hidden representation, a 512-dimensional feedforward layer, and dropout of 0.3. Early stopping uses validation loss with a patience of 10 epochs. Hyperparameters are selected only on the validation subset by grid search over 2/4/6 Transformer layers, hidden dimensions of 128/256/512, and dropout values of 0.1/0.3/0.5.

To evaluate ACG-CMT, we compare it with traditional machine-learning models, unimodal deep-learning models, and multimodal fusion baselines under the same learner-level data partition and evaluation protocol. Each model is trained and evaluated with five random seeds. Because five paired seed runs are too few for a reliable inferential comparison, and because an exact two-sided Wilcoxon signed-rank test with *n* = 5 has a highly discrete null distribution, the revised manuscript does not use the five seeds to claim statistical significance. The five runs are used only to characterize sensitivity to stochastic initialization, and performance is reported as mean ± standard deviation. We have removed the previously reported Wilcoxon *p*-values and significance asterisks. We have also removed naive window-level bootstrap confidence intervals, because overlapping windows violate the independence assumption of such resampling. If inferential uncertainty is required, resampling must respect the hierarchical data structure (preferably learner-level cluster resampling); no claim in this manuscript treats the 12,864 overlapping windows as independent replicates.

The ablation experiments focused on the key components of ACG-CMT, setting up variations such as removing the speech modality, removing the gesture and posture modality, removing the emotion modality, removing the cross-modal attention mechanism, removing the affective-cognitive gating mechanism, and removing the adaptive feedback decision module. These experiments were used to examine the impact of different modal information, cross-modal temporal modeling, dynamic gating fusion, and feedback decision tasks on model performance.

The evaluation metrics were set according to the task objectives of this paper. For English learning state identification, accuracy, precision, recall, and F1 score were used, with a focus on accuracy and F1 score; for affective-cognitive load estimation, R^2^, RMSE, and MAE were used, with a focus on R^2^ and RMSE; for feedback strategy matching, feedback strategy matching rate was used; and for system interaction efficiency, average feedback delay was used. These settings provide the experimental basis for the main results analysis, ablation experiments, affective-cognitive load estimation, feedback strategy matching, and interaction efficiency analysis in Section 4.

## 4 Results analysis

### 4.1 Analysis of the identification and comparison results of English learning status

This section conducts a comparative analysis of the English learning state recognition task, comparing the recognition performance of ACG-CMT with traditional machine learning models, single-modal deep learning models, and conventional multimodal fusion models on the test set. The analysis metrics selected are Accuracy, Macro-F1, AUC, and Difficult-state F1. The Difficult-state F1, composed of the F1 mean values of four abnormal learning states—pronunciation deviation, hesitation, comprehension difficulty, and low participation—reflects the model’s ability to recognize complex learning states. Different models are organized according to method category, input modality, and evaluation metrics; the results are shown in Table 2.

**Table 2:** Comparison results of English learning status recognition.

Model category	Model	Input modality	Accuracy	Macro-F1	AUC	Difficult-state F1
Traditional ML	SVM	Hand-crafted features	0.714 ± 0.005	0.691 ± 0.006	0.782 ± 0.007	0.673 ± 0.008
Traditional ML	Random forest	Hand-crafted features	0.742 ± 0.004	0.718 ± 0.005	0.811 ± 0.006	0.702 ± 0.007
Traditional ML	XGBoost	Hand-crafted features	0.768 ± 0.004	0.746 ± 0.005	0.836 ± 0.005	0.730 ± 0.006
Single-modal DL	Speech-BiGRU	Speech	0.756 ± 0.006	0.735 ± 0.007	0.827 ± 0.006	0.712 ± 0.008
Single-modal DL	Pose-ST-GCN	Gesture posture	0.733 ± 0.006	0.718 ± 0.007	0.801 ± 0.007	0.710 ± 0.008
Single-modal DL	Affect-MLP	Affective features	0.728 ± 0.007	0.716 ± 0.008	0.796 ± 0.008	0.709 ± 0.009
Multimodal fusion	Early-fusion transformer	Speech + Gesture + Affective	0.824 ± 0.006	0.809 ± 0.007	0.884 ± 0.006	0.798 ± 0.008
Multimodal fusion	Late-fusion transformer	Speech + Gesture + Affective	0.835 ± 0.005	0.819 ± 0.006	0.895 ± 0.005	0.809 ± 0.007
Multimodal fusion	Multimodal transformer	Speech + Gesture + Affective	0.861 ± 0.005	0.848 ± 0.006	0.918 ± 0.004	0.839 ± 0.007
Recent multimodal transformer	Cross-modal emotion fusion transformer	Speech + Gesture + Affective	0.869 ± 0.005	0.857 ± 0.006	0.923 ± 0.004	0.848 ± 0.007
Recent multimodal transformer	Noise-resistant multimodal transformer	Speech + Gesture + Affective	0.876 ± 0.004	0.866 ± 0.005	0.929 ± 0.004	0.857 ± 0.006
Proposed model	ACG-CMT	Speech + Gesture + Affective + Gate	0.892 ± 0.004	0.884 ± 0.005	0.941 ± 0.003	0.877 ± 0.006

All values are reported as mean ± standard deviation across five random-seed runs. These repetitions quantify sensitivity to stochastic initialization and are not treated as independent inferential replicates. No statistical-significance claim is made from *n* = 5 seed runs.

In Table 2, ACG-CMT achieves an accuracy of 0.892, a Macro-F1 of 0.884, an AUC of 0.941, and a difficult-state F1 of 0.877. The closest recent multimodal baselines remain lower on the reported point estimates. These differences are interpreted descriptively as consistent performance advantages under the fixed learner-level split, rather than as formal evidence of statistical significance. The standard deviations across the five seeds are small relative to the observed gaps, which indicates limited sensitivity to random initialization, but five seeds do not provide a sufficient basis for a reliable paired nonparametric significance test. Accordingly, the previously reported Wilcoxon *p*-values and significance language have been removed. The observed performance pattern is consistent with the contribution of cross-modal temporal modeling and state-dependent gating, while causal attribution to any single architectural component is reserved for the ablation analysis rather than inferred from *p*-values.

### 4.2 Ablation experiments and multimodal contribution analysis

This section conducts an ablation analysis to examine the impact of different components of ACG-CMT on model performance, comparing the complete model with variants that have had their speech modality removed, gesture/pose modality removed, emotion modality removed, cross-modal attention removed, affective-cognitive gating removed, and adaptive feedback module removed. The analysis metrics selected are Acc., F1, Load R^2^, Match, and ΔF1. Acc. and F1 represent performance in English learning state recognition, Load R^2^ represents performance in affective-cognitive load estimation, Match represents performance in feedback policy matching, and ΔF1 represents the decrease in Macro-F1 score of each ablation variant relative to the complete model. The ablation variants are organized according to the removed modules and corresponding task metrics, and the results are shown in Table 3.

**Table 3:** ACG-CMT ablation experiment and module contribution results.

Variant	Removed module	Acc.	F1	Load R^2^	Match	ΔF1
Full	—	0.892	0.884	0.836	0.861	0
w/o Speech	Speech	0.829	0.817	0.798	0.805	0.067
w/o Gesture	Gesture	0.846	0.833	0.807	0.823	0.051
w/o Affect	Affective	0.838	0.826	0.754	0.792	0.058
w/o CMA	Cross-modal attention	0.814	0.801	0.769	0.786	0.083
w/o AC-gate	Affective-cognitive gate	0.826	0.812	0.781	0.793	0.072
w/o Feedback	Feedback module	0.875	0.864	0.819	0.742	0.020

Acc. denotes the accuracy of learning state recognition; F1 denotes Macro-F1; Load R^2^ denotes the coefficient of determination for the affective-cognitive load estimation task; Match denotes the feedback strategy matching rate; ΔF1 denotes the decrease in Macro-F1 of each ablation variant compared with the full ACG-CMT model.

In Table 3, the F1 score of the complete ACG-CMT is 0.884. Removing cross-modal attention reduces F1 to 0.801 (ΔF1 = 0.083), removing the affective-cognitive gate reduces F1 to 0.812 (ΔF1 = 0.072), and removing the feedback module leaves F1 at 0.864 while reducing feedback matching to 0.742. These ablations indicate that cross-modal attention and state-dependent gating are important architectural contributors to predictive performance under the evaluated setting, whereas the feedback module primarily contributes to policy matching. The gating interpretation refers to latent affective/cognitive and pre-decision task context learned from inputs; it does not imply that the operational load reference-index target is supplied to the gate. As with the other internal analyses, the ablation results support an architectural contribution claim but not a causal psychological mechanism.

### 4.3 Analysis of affective-cognitive load estimation results

This section analyzes the results of the affective-cognitive load estimation task. Based on the operational affective-cognitive load reference-index values of the test set samples and the model prediction results, the visualization results are organized from three perspectives: prediction fit relationship, error consistency, and affective-cognitive interaction response. The figures show the affective-cognitive load estimation results using a scatter plot of predicted values versus reference-index values, the relationship between prediction error and the mean reference-index value using a Bland–Altman consistency analysis plot, and the relationship between affective intensity, expression instability, and predicted cognitive load under different task difficulties using affective-cognitive interaction response curves. The results are shown in Figure 5. All figures in this chapter, covering Figures 5–7, are generated from the prediction outputs, attention weights, and gating weights of the trained ACG-CMT on the test set.

**Figure 5:**
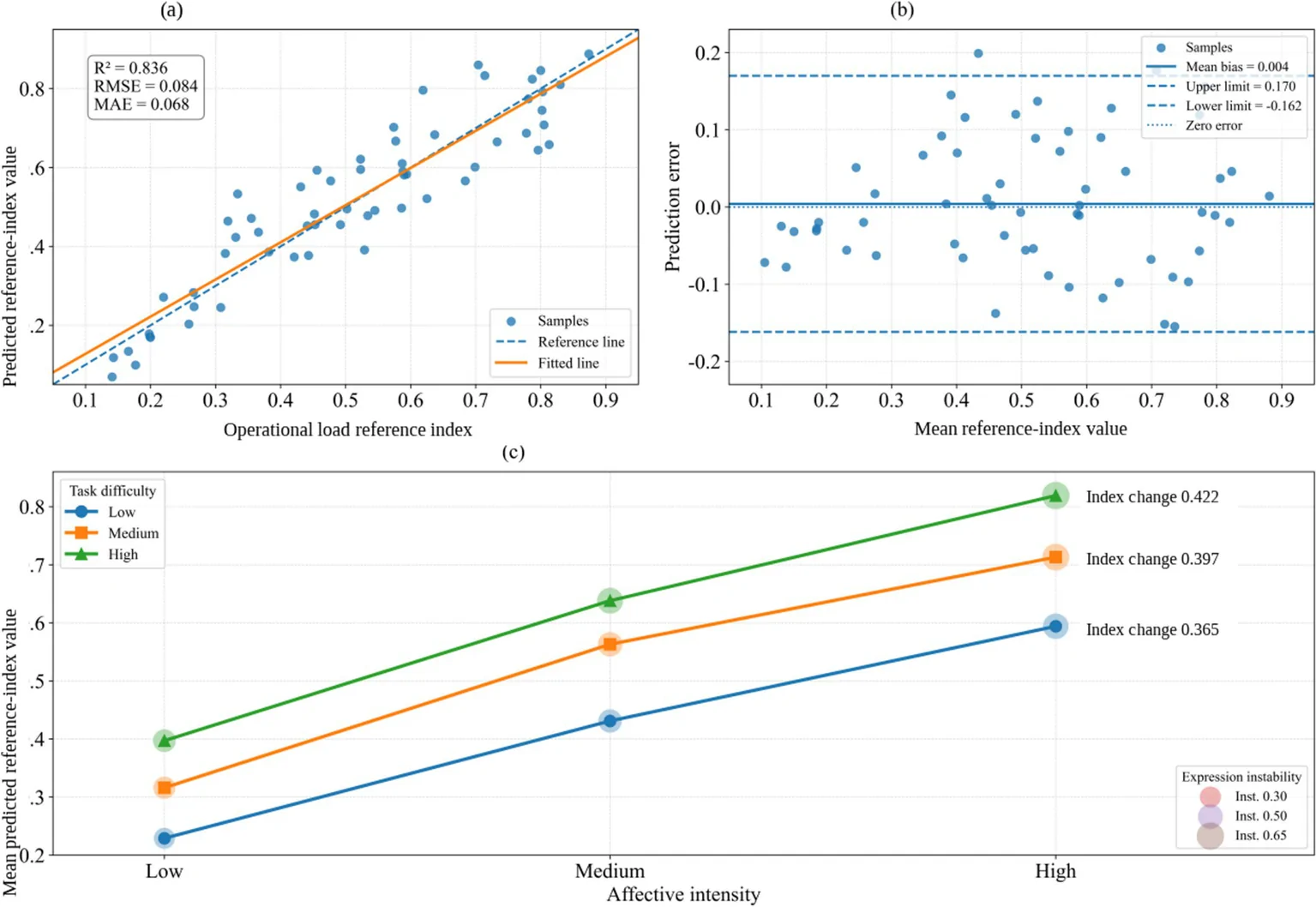
Relationship between affective-cognitive load estimation results and response. **(a)** Predicted values versus operational load reference index. **(b)** Bland–Altman agreement analysis. **(c)** Affective-cognitive interaction response curve.

In Figure 5, the affective-cognitive load estimation task yields R^2^ = 0.836, RMSE = 0.084, MAE = 0.068, with a mean prediction bias of 0.004 and Bland–Altman limits of agreement from −0.162 to 0.170. Across five random-seed runs, the standard deviation is 0.009 for R^2^ and 0.002 for RMSE, indicating low sensitivity to stochastic initialization. We do not report a naive window-level bootstrap confidence interval because the 50%-overlapping windows are correlated. The affective-cognitive interaction curve shows that the predicted composite load index increases with more demanding task conditions and stronger affective/behavioral difficulty signals. These associations support predictive consistency and model-level interpretability with respect to the operational reference index; they do not establish a biological, neurocognitive, or causal mechanism of cognitive load. Thus, the reported R^2^, RMSE, MAE, bias, and agreement limits quantify fidelity to the operational reference index; they do not constitute criterion validation of latent or physiological cognitive load.

### 4.4 Adaptive feedback strategy matching and interaction efficiency analysis

This section analyzes the results of adaptive feedback strategy matching and interaction efficiency. Based on teacher-annotated feedback, model-predicted feedback, feedback trigger time, and changes in learning state before and after feedback in the test set, it organizes visualization results at three levels: feedback intervention effect, effective feedback window, and multimodal precursor signals before feedback triggering. The figure uses the state recovery gain and load reduction after feedback to present the intervention results of different feedback strategies; the matching rate and state recovery gain under the delay window to present the feedback timeliness; and the pause ratio, emotional intensity, gesture instability, and load risk trajectory before feedback triggering to present changes in feedback precursors. The results are shown in Figure 6.

**Figure 6:**
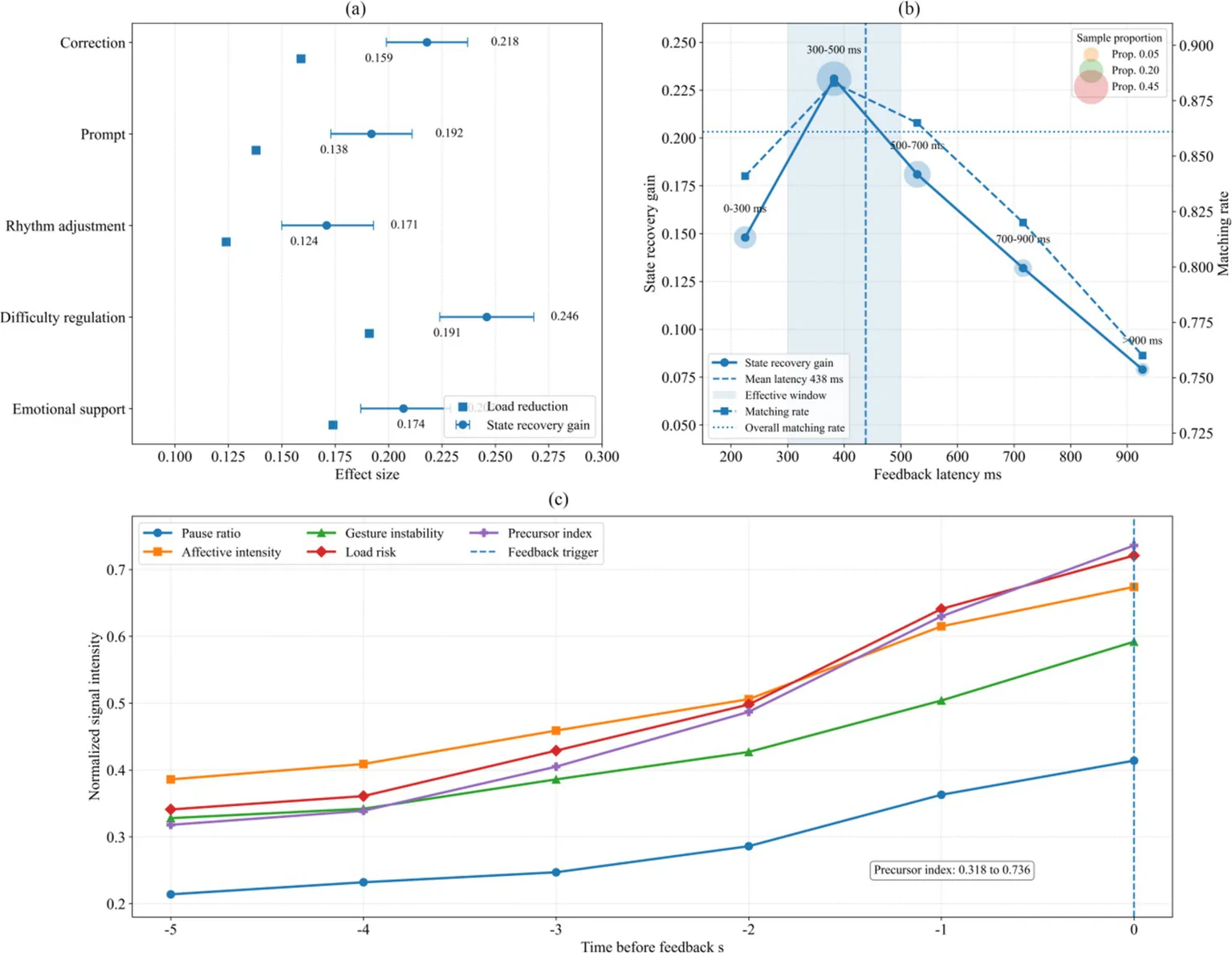
Effectiveness of adaptive feedback strategy—time window—pre-feedback precursor signals. **(a)** Feedback effectiveness after intervention. **(b)** Effective feedback time window. **(c)** Pre-feedback precursor signals.

In Figure 6, the overall feedback-strategy matching rate is 0.861 and the average feedback delay is 438 ms. Across five random-seed runs, the standard deviation is 0.006 for matching rate and 17 ms for feedback delay. A naive window-level bootstrap confidence interval is not reported because overlapping windows are correlated. Within the 300–500 ms feedback window, the matching rate reaches 0.883 and the state-recovery gain reaches 0.231, compared with 0.148 for 0–300 ms and 0.079 for >900 ms. From 5 s before feedback triggering to the trigger time, the precursor index increases from 0.318 to 0.736, pause ratio from 0.214 to 0.414, and load-risk estimate from 0.341 to 0.721. These results show an association between multimodal precursor patterns, teacher-labeled feedback selection, and timing; they should not be interpreted as causal proof that the detected precursor signals produce the subsequent recovery.

### 4.5 Internal representation and interpretability analysis of the model

This section analyzes internal representation patterns and *post hoc* interpretability of ACG-CMT in learning-state recognition and feedback decision-making. The analysis summarizes cross-modal attention responses, affective-cognitive gating weights, robustness under controlled noise perturbation, and representation changes across task types. These analyses are intended to show how the trained model reallocates representational emphasis under different observed states; they do not identify a causal psychological mechanism or establish that attention/gating weights are direct explanations of human cognition.

In Figure 7, the gate weights vary systematically across observed learning states: under normal expression the three modalities remain relatively balanced (0.320–0.350), whereas the speech weight increases under pronunciation deviation, the affective weight increases under comprehension difficulty, and the gesture weight increases under low participation. Under joint speech-gesture noise, Macro-F1 decreases from 0.884 at 20 dB/zero gesture noise to 0.694 at 0 dB/0.5 gesture noise, and open-ended oral Q&A shows a larger representation shift than reading-aloud tasks. These findings indicate that the trained model’s internal representations are state- and context-dependent and that the gating trajectories offer a useful *post hoc* description of modality reliance. However, correspondence between model weights and observed learner states is associative rather than mechanistic: it does not demonstrate causal coupling among pronunciation difficulty, bodily hesitation, emotion, and cognition, nor does it constitute direct evidence for embodied-cognition theory. The patterns are instead described as being consistent with an embodied multimodal interpretation and as motivating future validation with independent behavioral and physiological measurements.

**Figure 7:**
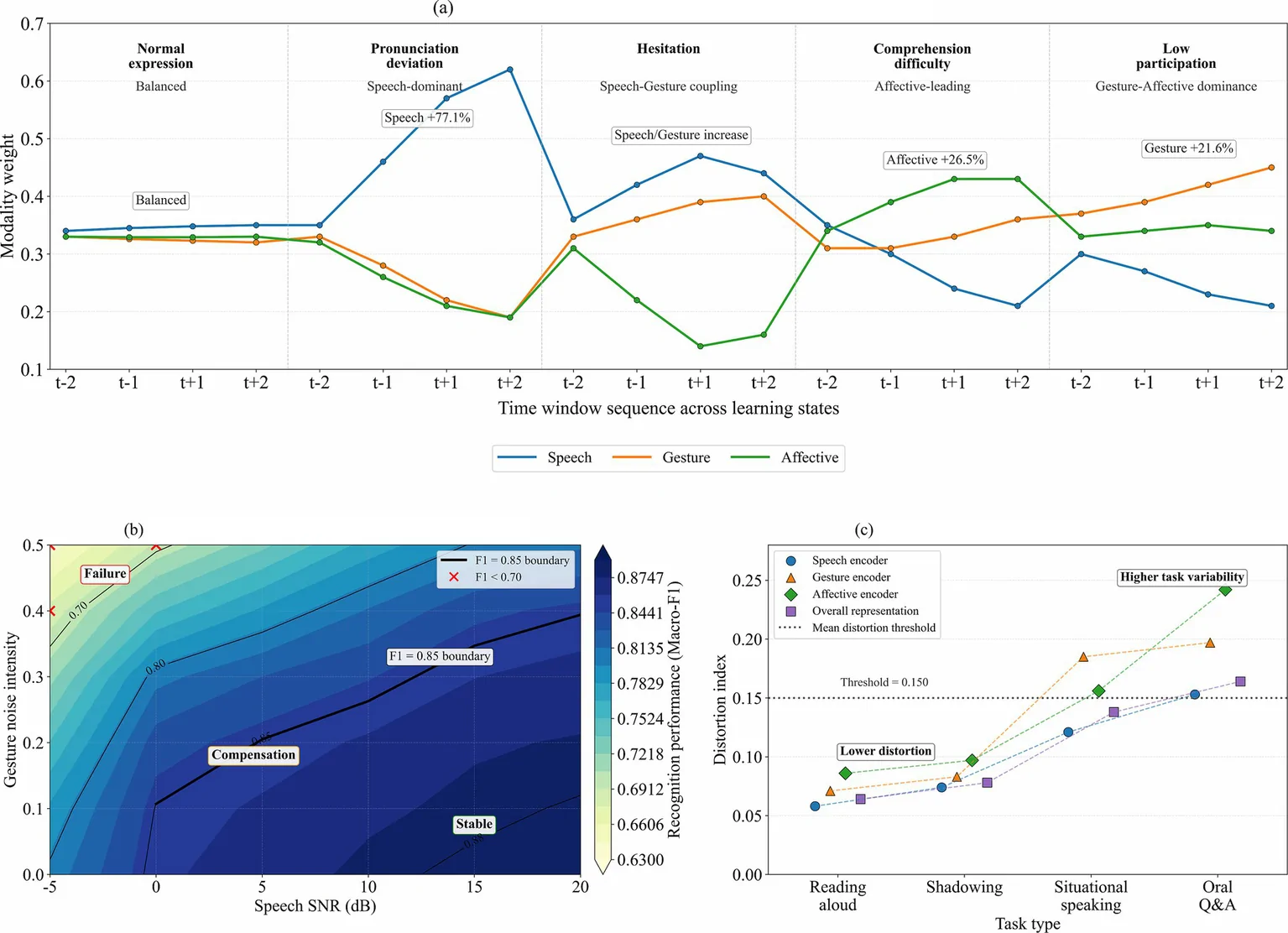
Analysis of the internal mechanism and generalization performance of the ACG-CMT model. **(a)** Continuous modal gating trajectories under different learning states. **(b)** Robustness contour lines under joint speech-gesture noise. **(c)** Comparison of representation distortion under different task types.

## 5 Conclusion

This study proposed the Affective-Cognitive Gated Cross-modal Transformer (ACG-CMT) to address limitations in multimodal perception, affective-cognitive state characterization, and adaptive feedback regulation in English language learning systems. ACG-CMT integrates speech, gesture, and affective information through modality-specific encoding branches, cross-modal attention, and state-dependent gating to construct a unified representation for learning-state recognition, affective-cognitive load estimation, and feedback policy matching. Experimental results show that the proposed model achieves an accuracy of 0.892 for English learning-state recognition, an R^2^ of 0.836 for affective-cognitive load estimation, and a feedback policy matching rate of 0.861. These results indicate that ACG-CMT provides relatively stable performance across learner-state discrimination, load estimation, and adaptive feedback decision-making.

By jointly modeling linguistic performance, embodied behavior, and affective responses, the proposed framework provides a computational basis for identifying pronunciation difficulties, detecting hesitation and comprehension-related problems, estimating learner load, and determining appropriate feedback timing during oral English learning activities. More broadly, the unified multimodal representation enables intelligent language learning systems to move beyond outcome-oriented assessment toward process-level perception, learner-state interpretation, and adaptive interaction. The state-dependent gating mechanism further provides interpretable information about how the relative contributions of speech, gesture, and affective cues vary across different learning conditions.

Several limitations should nevertheless be acknowledged. First, the term affective-cognitive is used because the framework models affective responses and an operational composite load index from observable multimodal behavior; no neurophysiological signals were collected, so the present results should not be interpreted as direct evidence of neural or neurocognitive mechanisms. Second, the composite load reference combines NASA-TLX with reaction time, pause ratio, and task difficulty using expert weighting. This provides a pragmatic multi-indicator supervision signal, but the weighting scheme has not been independently criterion-validated against physiological measurements; future work should preregister and sensitivity-test alternative weighting schemes and compare the index with independent measures such as pupillometry or other validated workload indicators. Consequently, the present composite should not be interpreted as a validated ground truth, and no claim of criterion validity is made. Third, the 5-s windows overlap by 50%, producing within-segment correlation. Learner-level splitting prevents train-test leakage, but the 12,864 windows are not 12,864 independent statistical observations, and inferential uncertainty should be estimated with cluster-aware procedures. Fourth, the participant sample consisted primarily of non-English-major undergraduate students, limiting generalizability to other ages, educational backgrounds, and proficiency levels. Fifth, the experiments were conducted mainly under controlled learning tasks and have not yet been extensively evaluated in long-term, open-ended, naturally occurring learning environments.

Future research should therefore validate ACG-CMT with more diverse learner populations and across broader language-learning contexts, including authentic online interaction, collaborative learning, and longitudinal instructional settings. Further work may also examine cross-domain generalization, robustness under naturally occurring multimodal noise, and the long-term pedagogical effects of adaptive feedback. Such extensions would provide stronger evidence regarding the practical applicability, generalizability, and sustained educational value of embodied multimodal interaction frameworks in intelligent language learning systems.

## Data Availability

The original contributions presented in the study are included in the article/supplementary material, further inquiries can be directed to the corresponding author.
